# Benefit of Action

**Published:** 1854-04

**Authors:** 


Benefit of Action.—So far from complete inaction being perfect enjoyment, there are few greater sufferings than that which the total absence of occupation generally induces. Count Caylies, the celebrated French antiquary, spent much time in engraving the plates which illustrated his valuable work. When his friends asked him why he worked so hard at such an almost mechanical occupation, he said—"*Je grave pour ne pas me pendre*"—I engrave lest I should hang myself. When Napoleon was slowly withering away, from disease and *ennui* together, on the rock of St. Helena, it was told him that one of his old friends, an ex-colonel in the Italian army, was dead. "What disease killed him?" asked Napoleon. "That of having nothing to do," it was answered. "Enough," said Napoleon, "even had he been an emperor."

